# Highly Sensitive Detection of *Staphylococcus aureus* Directly from Patient Blood

**DOI:** 10.1371/journal.pone.0031126

**Published:** 2012-02-17

**Authors:** Padmapriya P. Banada, Soumitesh Chakravorty, Darshini Shah, Michele Burday, Fermina M. Mazzella, David Alland

**Affiliations:** 1 Division for Infectious Diseases, Department of Medicine, New Jersey Medical School – University of Medicine and Dentistry of New Jersey, Newark, New Jersey, United States of America; 2 Department of Pathology and Laboratory Medicine, New Jersey Medical School – University of Medicine and Dentistry of New Jersey, Newark, New Jersey, United States of America; Universidade Federal de Minas Gerais, Brazil

## Abstract

**Background:**

Rapid detection of bloodstream infections (BSIs) can be lifesaving. We investigated the sample processing and assay parameters necessary for highly-sensitive detection of bloodstream bacteria, using *Staphylococcus aureus* as a model pathogen and an automated fluidic sample processing – polymerase chain reaction (PCR) platform as a model diagnostic system.

**Methodology/Principal Findings:**

We compared a short 128 bp amplicon hemi-nested PCR and a relatively shorter 79 bp amplicon nested PCR targeting the *S. aureus nuc* and *sodA* genes, respectively. The *sodA* nested assay showed an enhanced limit of detection (LOD) of 5 genomic copies per reaction or 10 colony forming units (CFU) per ml blood over 50 copies per reaction or 50 CFU/ml for the *nuc* assay. To establish optimal extraction protocols, we investigated the relative abundance of the bacteria in different components of the blood (white blood cells (WBCs), plasma or whole blood), using the above assays. The blood samples were obtained from the patients who were culture positive for *S. aureus*. Whole blood resulted in maximum PCR positives with *sodA* assay (90% positive) as opposed to cell-associated bacteria (in WBCs) (71% samples positive) or free bacterial DNA in plasma (62.5% samples positive). Both the assays were further tested for direct detection of *S. aureus* in patient whole blood samples that were contemporaneous culture positive. *S. aureus* was detected in 40/45 of culture-positive patients (sensitivity 89%, 95% CI 0.75–0.96) and 0/59 negative controls with the *sodA* assay (specificity 100%, 95% CI 0.92–1).

**Conclusions:**

We have demonstrated a highly sensitive two-hour assay for detection of sepsis causing bacteria like *S. aureus* directly in 1 ml of whole blood, without the need for blood culture.

## Introduction

Blood stream infections (BSIs) are a serious cause of morbidity and mortality worldwide. Early diagnosis and appropriate treatment of sepsis has been shown to decrease life-threatening complications [1], [2], [3], [4], [5]. BSIs are usually diagnosed by performing a series of blood cultures [1]. Unfortunately, this technique is slow, usually requiring one or more days to produce a positive result. Nucleic acid-based amplification tests (NAATs) are potentially more rapid and sensitive than blood cultures [6], [7], [8], [9]. However, even NAATs appear to have limited sensitivity when applied to detecting bacteria directly from patient blood samples. For this reason, most NAATs require bacteria to be enriched in blood cultures before the cause of a BSI can be reliably detected [10], [11]. One commercially available NAAT, the SeptiFast™ assay (Roche Diagnostics, Indianapolis, IN), appears to adequately detect BSI directly from non-enriched blood samples. However, the SeptiFast™ assay requires a total of 6 hours to complete, including 3 hours of technician hands-on time [12]. Furthermore, the overall bacterial detection rate among suspected sepsis patients was relatively poor and sensitivity varied highly among different studies [13], [14], [15].

Most adult patients with BSI have relatively low concentrations of bacteria in their blood [16]. Blood is also highly inhibitory to the polymerase chain reaction (PCR) [17], [18]. NAAT-based BSI assays must therefore be highly sensitive; and the nucleic acid extraction protocols which are used must recover as much bacterial DNA as possible from a relatively large volume of blood. Extraction techniques must also ensure that the DNA which is recovered is inhibitor-free and of high purity. These requirements increase the cost and complexity of NAAT-based BSI assays. The development of the GeneXpert® sample processing and PCR system (Cepheid, Sunnyvale, CA) suggested some simple solutions to these issues. The GeneXpert® system uses a filter-capture approach to isolate bacteria from relatively large blood volumes. The internal fluidics of the system's sample processing cartridge permits extensive sample washing. Finally, a multi-chamber cartridge design makes it possible to increase assay sensitivity through the use of nested PCR without the risk of amplicon cross contamination.

Several critical questions need to be resolved before sample processing and amplification systems can be used to optimally detect BSI directly from patient blood. First, what analytic sensitivity is required to detect most cases of BSI? Prior studies examining the average number of bacterial colony forming units (CFU) per ml of blood in BSI patients cannot directly answer this question since DNA from non-viable bacteria may also be present in blood. Second, what blood components contain the maximum signal for BSI? The DNA target of a PCR assay could presumably be present in blood as free plasma DNA, or it could be present within intact (viable or non-viable) bacteria that are either freely floating in blood or concentrated within WBC. Extraction protocols would clearly vary depending on the requirements to capture DNA from these different blood compartments. To our knowledge, no study has yet examined the blood of BSI patients to see which blood compartment contains the maximum number of bacterial DNA targets for a PCR assay. The answers to these questions are of critical importance for a good assay design.

*Staphylococcus aureus* is responsible for a significant proportion of BSIs [19], [20]. In this study, we have used *S. aureus* as a model pathogen to investigate assay and blood processing parameters required to achieve maximal sensitivity for NAAT-based BSI detection. In the process, we designed and tested a highly sensitive assay to detect *S. aureus* directly from patient blood that used a customized GeneXpert® platform. To our knowledge, this is the first study to systematically determine the presence of bacteria in different blood components. This is also the first time a completely automated hands-free integrated sample processing and PCR based detection method has been successfully demonstrated to detect *S. aureus* directly in patient blood samples, without the need for culture.

## Methods

### Ethics statement

The study was approved by University of Medicine and Dentistry of New Jersey (UMDNJ)-Institutional review board (IRB) with the IRB protocol number: 0120080060 as “exempt” #4 because the tested samples were de-identified and would normally have been discarded. Informed consent of the study subjects was not required due to the nature of the samples.

### Preparation of bacterial cells and genomic DNA for analytical experiments

*S. aureus* ATCC 25923 was used for all analytic studies. The initial inoculum was prepared by growing the bacteria at 37°C in Luria-Bertini (LB) broth (BD, Sparks, MD) for 16–18 h. Serial dilutions were made in LB broth and plated on brain heart infusion (BHI) agar to evaluate the colony forming units (CFU) per milliliter; and the same dilutions were used in analytical experiments. Genomic DNA from *S. aureus* was purified using the GenElute bacterial genomic DNA kit (Sigma-Aldrich, St. Louis, MO). Genomic DNA from all bacteria tested for specificity was isolated by boiling at 90°C for 15 min using InstaGene Matrix reagent (Biorad, Hercules, CA). The DNA in the supernatant was transferred to a fresh tube and stored at −20°C until further use.

### Optimization of the PCR assays

Two hemi-nested PCR assays were developed to test *S. aureus* detection, a “short amplicon” (100–200 bp) assay targeting the *S. aureus nuc* gene and a “shorter amplicon” (<100 bp) assay targeting the *S. aureus sodA* gene. Gene specific primers were designed using the PrimerSelect (DNASTAR Lasergene ver 8.1.3) and/or Primer3 [21] programs and the molecular beacons to the inner PCR fragment were designed using the Mfold web server [22] (Data S1). Real-time nested amplification targeting *nuc* and *sodA* gene was performed in a GeneXpert® cartridge controlled by GeneXpert® instrument. For the *nuc* assay, the hemi-nested PCR reaction consisted of two sets of primers designed to sequentially amplify an outer 182 bp and an internal 128 bp amplicon (Data S1). For the *sodA* assay, the nested PCR reaction consisted of two sets of primers designed to sequentially amplify an outer 161 bp and an inner 79 bp amplicon. For both assays, the inner PCR reaction mix consisted of 1× PCR buffer (10 mM Tris-HCl, pH 8.3, 50 mM KCl and 0.001% (w/v) gelatin) with 4 mM MgCl_2_, 250 µM of each nucleotide (deoxynucleoside triphosphate), 0.5 µM of each primer (forward and reverse), 4 ng of the molecular beacon and 4 U of Jumpstart *Taq* DNA polymerase (Sigma Aldrich, St. Louis, MO). The outer PCR reaction mix consisted of the same components, except for MgCl_2_ which was added at 3 mM and 3.5 mM concentrations for the *nuc* and *sodA* PCR assays, respectively. The outer assays also omitted the molecular beacons. The outer PCR was performed in a final volume of 80 µl, which included 1 µl of genomic DNA or 40 µl of bacterial lysate, depending on the target being amplified. PCR was performed using the thermal cycling conditions described in Data S1. To examine the effect of amplicon size on the LOD, a second set of *sodA* primers were designed to amplify a 79 bp region within the 161 bp *sodA* amplicon (Data S1). The primers were designed to have a Tm similar to the primers amplifying the larger amplicon. The two *sodA* assays were compared using identical PCR conditions (Data S1) without nesting.

### Collection and storage of clinical patient blood samples

To obtain blood samples from patients with bacteremia, k2-EDTA anticoagulated blood tubes submitted for complete blood counts (CBCs) to the Department of hematology at the University of Medicine and Dentistry of New Jersey (UMDNJ) University Hospital (UH) were saved in a refrigerator at 4°C for 4–6 days after they had been used for routine purposes and would normally have been discarded. These tubes usually had 1 to 4 ml of blood remaining. With the help of the UH clinical microbiology laboratory, patients with *S. aureus* positive blood cultures were identified and matched with the tubes stored at the UH hematology laboratory based on patient identification numbers. Patient identifiers were removed before the samples were brought to the research laboratory for testing by the GeneXpert system. Culture-negative control samples were similarly collected after confirming that the patient had not had positive blood cultures during the CBC blood collection period. For use in analytical experiments, culture-negative k2-EDTA blood was collected as described above and blood from individual tubes was pooled to obtain larger volumes. An aliquot was tested by PCR to confirm the absence of detectible *S. aureus*. The study was approved by the UMDNJ Institutional review board.

### Blood sample processing

**Figure 1:**
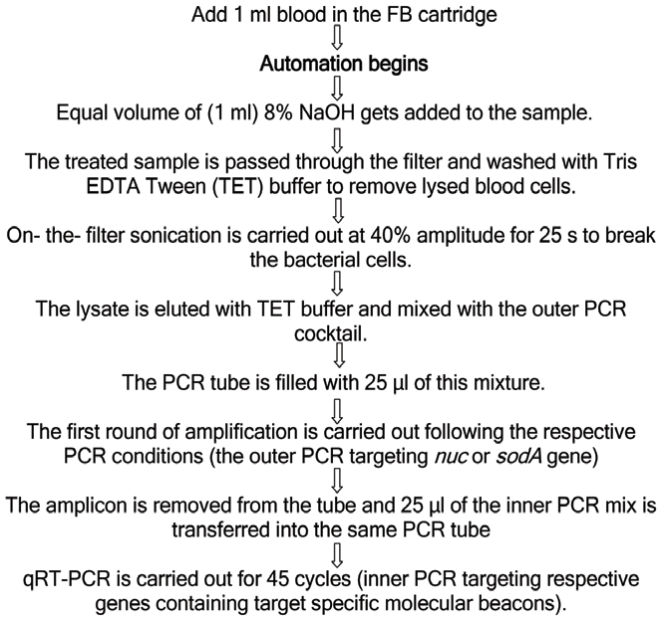
Flow diagram of the fluidic steps involved in sample processing and PCR amplification for target genes.

For whole blood studies, one ml of CBC blood was processed in a GeneXpert® cartridge containing a filter (the filter-based (FB) cartridge) to capture intact bacteria from clinical samples, as follows. Different chambers within empty FB cartridges were manually filled with PCR reaction mix or sample processing buffers. A blood sample was added to a FB cartridge's sample chamber, and the cartridge was then inserted into a GeneXpert® system module. The sample was then processed within the cartridge by the GeneXpert® system according to a specified protocol using customized fluidics software. The automated sample processing steps of the assay protocol are shown in Figure 1. A link showing the fluidics of a similar assay is available at http://www.youtube.com/watch?v=HF6HzlqOhkg. The blood sample was first mixed with 1∶1 8% NaOH; the lysed blood was then passed through the internal cartridge filter; the filter-captured bacteria were then extensively washed with a Tris (50 mM)- EDTA (0.1 mM) –Tween (0.1%) (TET) buffer, pH 8.4; glass beads present in the filter chamber were then agitated by an ultrasonic horn to lyse the bacterial cells. The liberated DNA was washed through the filter into a collection chamber and then mixed with the outer PCR mix. For a 1 ml blood sample, all of the bacteria present in the sample were captured on the FB cartridge filter. After cell lysis, bacterial DNA was eluted-off of the filter and mixed approximately 1∶1 with PCR reagents. Approximately 30% of the total eluted DNA was then moved into the PCR tube which is integrated into the FB cartridge for PCR amplification and detection. PCR was then performed as described above. To perform nested PCR, the first PCR reaction included a step where the outer PCR amplicon was withdrawn from the integrated PCR and approximately 10% was then mixed with the inner PCR buffer. Real-time PCR of the inner amplicon was performed. A positive signal was indicated by the real-time cycles vs fluorescence units curve for the selected fluorophore. The cycle threshold (Ct) was calculated by the GeneXpert® software.

### Analytical sensitivity and specificity tests

Analytical sensitivity of both the short amplicon *nuc* PCR and the shorter amplicon *sodA* PCR assays was determined using genomic DNA and bacterial cells. Genomic DNA copy number was estimated based on the molecular weight of the *S. aureus* genome (2.7–2.8 mbp) using the copy number calculator on URI Genomics and Sequencing Center website (http://www.uri.edu/research/gsc/resources/cndna.html), and test sample were created by diluting stock DNA in water.

To determine the limit of detection of *S. aureus* in whole blood, the cells were prepared from pre-quantified cell stock and serially diluted as explained before. Serial dilutions were made in LB broth and spiked into culture negative whole blood to achieve cell concentrations ranging from 1 CFU/ml through 100 CFU/ml. One ml of this sample was loaded into chamber 3 of the FB cartridge. The *nuc* and *sodA* assays were performed side-by-side to compare assay performance in replicates of six per target concentration. The assay was scored based on the number of positive assays per target concentration.

Assay specificity was examined in the GeneXpert system as a nested reaction by spiking outer PCR mix with 1 ng of genomic DNA from coagulase negative staphylococci (*S. epidermidis, S. hominis, S. capitis, S. cohnii, S. haemolyticus, S. hominis, S. lugdunensis, S. sciuri, S. schleiferi, and S. warneri), Escherichia coli, Klebsiella pneumoniae, and Serratia marcescens* clinical isolates. Assay specificity was further assessed by testing the outer and inner primers separately in PCR reactions of DNA extracted from *Acinetobacter baumanii, Bacillus subtilis, B. cereus, Campylobacter jejuni, Citrobacter freundii, Corneybacterium pseudodiptheriae, Enterobacter cloacae, enterococcus avium, E. faecalis, E. faecium, Klebsiella oxytoca, Haemophilus influenzae, Proteus mirabilis, P. vulgaris, Streptococcus agalactiae, S. pneumoniae* and *Staphylococcus epidermidis* using the LightCycler480 (Roche, Indianapolis, IN) as a detection system. All samples were obtained from UMDNJ's UH Microbiology laboratory. *S. aureus* ATCC 25923 genomic DNA was used as positive control in these studies.

### Relative bacterial abundance in different blood components

**Figure 2:**
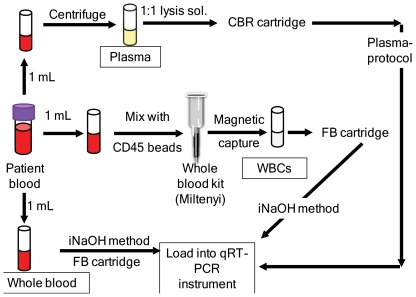
Blood components study processing schematic. CBC blood from patients with blood culture positive for *S. aureus* was divided into 1 ml each for detection of *S. aureus* load in plasma, white blood cells (WBCs) and whole blood. Plasma was processed using a column based resin (CBR) cartridge. WBCs and whole blood was processed in a filter based (FB) cartridge.

BSI-causing bacteria can theoretically exist in three blood components: 1) as intact bacteria that are free within patient blood, 2) as free DNA in patient plasma (presumably from lysed bacteria), or 3) as intact bacteria that are trapped in WBCs. The utility of a particular sample processing technique to detect BSI may depend greatly on the relative amounts of target in each of these components. Patient blood (in k2-EDTA tubes) samples, which were culture positive for *S. aureus* were obtained as described above. Most CBC tubes used in this experiment contained a total of 3–4 ml blood. These tubes were divided into three fractions of 1 ml each and any remaining blood was discarded or used for other experiments. One fraction was processed for plasma isolation, the second for WBC isolation and the third was used as whole blood (Figure 2). An occasional CBC tube contained only 2 ml of blood. In this case, the blood was divided into 2 ml fractions and two out of the three blood components were tested. To test for intact bacteria in whole blood, one ml was processed in the FB cartridge as described above. To test for free bacterial DNA in plasma, one ml of blood was centrifuged at 1200× g for 15 min and the supernatant was transferred to a sterile Eppendorf® tube. The supernatant was passed through a 0.45 µ syringe filter (Corning Inc, Corning, NY) and the filtrate (350–500 µl) was collected in a sterile tube [23], to avoid carry-over of any WBCs or free cells. The volume was then increased up to 700 µl with sterile phosphate buffered saline (PBS, pH 7.4) and mixed 1∶1 with lysis buffer (containing 1∶1 ethanol and 5 M GTC in Tris buffer, pH 8.0) and a bead (0.75 µg) of proteinase K. The mix was vortexed well and the whole (1400 µl) preparation was added to the sample loading slot in a column based resin (CBR) cartridge, which is designed to capture free DNA rather than intact cells. The captured DNA was eluted with Tris EDTA Tween (TET) buffer.

To test for bacterial cells in WBC, WBCs were extracted from one ml of blood using a whole blood column in conjugation with CD45+ microbeads (Miltenyi biotech, Auburn, CA). The labeling and isolation of WBCs were carried out following the manufacturer's recommendations. Also as recommended, the bound WBCs were extracted from the column in 5 ml of blood separation buffer. The total cells were evaluated microscopically by mixing a 100 µl of this opaque-white elute with 100 µl of trypan blue and 10 µl was loaded on to a haemocytometer (Reichert, Buffalo, NY). No significant concentration of red blood cells (RBC) was observed. The whole 5 ml of WBCs were centrifuged at 1200× g for 15 min [24], the supernatant was discarded and the pellet was resuspended in 1 ml of sterile PBS and then processed in the Genexpert® system using the same software protocol as for whole blood (iNaOH) (Figure 2). A cycle threshold (Ct) cut-off of 40 was set based on the analytical LOD experiments.

### Detection of *S. aureus* in patient blood samples

Whole blood from a total of 96 patients confirmed to be positive for *S. aureus* by blood culture, were selected and stored as described before. One-ml of the blood sample was tested directly in the GeneXpert FB cartridge system, using both *nuc* and *sodA* assay following the iNaOH blood sample processing methodology described in Figure 1.

### Statistics

Standard statistical analysis (average, standard deviation and t-test) was performed using Microsoft Excel 2000 for Windows. Confidence interval levels were calculated using the online clinical calculator 1 available on the website http://faculty.vassar.edu/lowry/clin1.html. Linear regression model to fit percent positives curves was performed using GraphPad Prism ver 5.04 for windows, GraphPad software, San Diego, California, USA, www.graphpad.com.

## Results

### Analytical sensitivity and specificity

**Figure 3:**
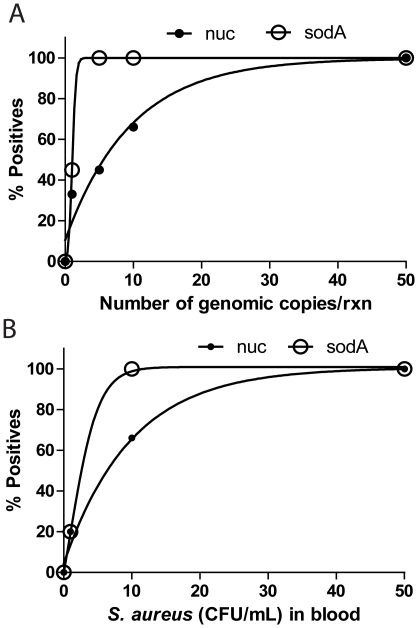
Analytical sensitivity of the *nuc* and *sodA* assays for the detection of *Staphylococcus aureus* genomic DNA (A) and *S. aureus* cells spiked in blood (B).

Many NAAT detection assays are considered highly-sensitive if they have a limit of detection (LOD) of less than 50 targets per ml [25]. We postulated that even this high degree of sensitivity might not be sufficient to detect BSI directly from patient blood. Two different assays targeting the *nuc* gene, a hemi-nested PCR producing a short amplicon (128 bp), and a second assay targeting *sodA* in a fully nested PCR with a shorter amplicon (79 bp) were tested analytically. In tests of genomic DNA (Figure 3A), both assays could detect 50 genomic copies per reaction 100% of the time; however, the *sodA* assay was clearly more sensitive as it could detect 5 genomes 100% of the time versus 60% of the time for the *nuc* assay. The *sodA* assay was also confirmed to be more sensitive in tests using *S. aureus* cells spiked into blood (Figure 3B). The increased sensitivity of this assay is likely due to its very small amplicon size, since a *sodA* assay targeting the same region but producing larger amplicons similar to the *nuc* assay had a decreased limit of detection similar to the more poorly-performing *nuc* assay (Data S2). Both *nuc* and *sodA* nested PCR assays were 100% specific when tested against genomic DNA from all of the control bacteria mentioned in the materials and methods section.

### Relative bacterial abundance in different blood components

**Figure 4:**
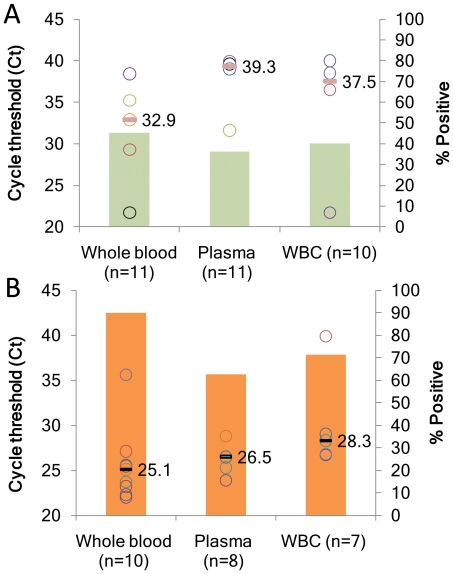
Detection of bacterial load in different blood components by *nuc* (A) and *sodA* (B) assays. Each Ct data point (open circles) indicates individual patient samples tested in each blood component (one color for each patient sample). The horizontal line indicates median Ct values. Solid color bars in green (*nuc* assay) and orange (*sodA* assay) represent the corresponding % positive on the secondary axis. An “n”, indicates the number of samples analyzed. WBC, indicates white blood cells.

We investigated the relative distribution of bacteria in three blood components (whole blood, WBC and plasma) in patients with *S. aureus* BSI using the *nuc* and *sodA* assays. Patient blood samples (in K2-EDTA tubes) submitted for complete blood count (CBC), who had a contemporaneous blood sample that was culture positive for *S. aureus* were selected and fractionated into each of the three blood components [or randomly into two components if sufficient blood volume (≥3 ml) was not available]. The components were then tested either with the *nuc* (Figure 4A) or *sodA* assay (Figure 4B) to determine whether *S. aureus* could be detected, and to compare the relative amounts of target in each component. Samples with Ct>40 were considered negative.

In our studies, we found that intact bacteria present in whole blood accounted for the largest number of samples that were positive for *S. aureus* (45% tested with the *nuc* assay and 90% tested with the *sodA* assay). Intact bacteria within WBCs were the next most likely to test positive (40% and 71%, respectively with *nuc* and *sodA* assays), while free DNA in plasma was the least likely to test positive (36% and 62.5%, respectively). Importantly, the whole blood fraction also produced earlier Ct values for positive assays than the other components, with median Ct values of 32.9, 39.3 and 37.5 for whole blood, WBCs and plasma respectively, with the *nuc* assay (Figure 4A). Similarly, the more sensitive *sodA* assay produced median Ct values of 25.1 for whole blood compared to 26.5 for WBCs and 28.3 for plasma (Figure 4B).

Our whole blood components extraction protocol recovered intact bacterial cells present within WBCs as well as free bacteria that were not cell-associated. We observed higher percent positives in whole blood compared to WBCs. Subtracting the median ct value from the whole blood samples from that of WBCs yielded a difference of 4.6 median Ct values (with *nuc* assay) indicating that >10 times more intact bacteria were present in blood outside of WBCs than within WBC. We also observed higher percent positives in whole blood compared to WBCs. These results strongly suggest that bacterial DNA largely exists in intact extracellular bacteria in patients with *S. aureus* BSI. Sample processing protocols that focus on extracting DNA from these intact bacteria in whole blood are likely to be the most successful.

### BSI detection in patient blood samples

Our blood components studies strongly suggested that the Cepheid FB cartridge was well-suited for recovering *S. aureus* from the blood of patients with *S. aureus* BSI. We used this system to examine the performance of the *nuc* and *sodA* assays for *S. aureus* BSI detection compared to blood culture. A total of 96 k2-EDTA blood samples from patients with *S. aureus* positive blood cultures and 88 k2-EDTA blood samples from patients who were culture-negative for *S. aureus* were collected. Fifty-one positive and 29 negative samples were tested with the *nuc* assay; and 45 positive and 59 negative samples were tested with the *sodA* assay (Table 1). The *nuc* assay detected *S. aureus* in 29/51 (sensitivity 57%, 95% CI 0.42–0.7) patients with *S. aureus* BSI and excluded *S. aureus* in 28/29 who did not have a documented *S. aureus* BSI (specificity 96.5%, CI 0.88–0.99), including 15 patients who were culture positive for other bacterial species. The one nuc positive sample had a Ct value of 36 and was confirmed to have *S. aureus* by DNA sequencing.

**Table 1:** Sensitivity and specificity of *nuc* and *sodA* assays compared to blood culture as tested with patient blood samples.

Assay	Number of positives	
	Culture positive for *S. aureus* (% Positive rate)	Culture negative for *S. aureus* (% Specificity)
*Nuc*	29/51 (57%)	1/29 (97%)
*SodA*	40/45 (89%)	0/59 (100%)

The *sodA* assay performed significantly better than the *nuc* assay (P<0.05). The assay was positive in 40/45 (89%, 95% CI 0.75–0.96) blood samples of patients with *S. aureus* BSI and negative in 59/59 (100%, 95% CI 0.92–1) patients without *S. aureus* BSI including 20 patients who were culture-positive for other bacterial species. Overall the *sodA* assay detected 32% more cases of *S. aureus* BSI than the *nuc* assay (p<0.05). These results indicate that efficient BSI detection requires assays with an analytic sensitivity of <10 CFU per ml and when combined with the GeneXpert FB cartridge sample processing system, can result in very high case detection rates.

## Discussion

This study investigates the assay parameters required to sensitively detect target bacteria in patients with *S. aureus* BSI. We discovered that both intact extracellular and intracellular bacteria in blood contribute to the majority of the positive assay signal. Extraction protocols must target these bacteria for optimal sensitivity. Protocols that retain WBCs or extract free DNA from plasma are unlikely to further increase assay sensitivity. This finding is important because WBCs contain human genomic DNA which may inhibit PCR assays [18], [26]. Free plasma DNA may be quite complex and laborious to extract.

We also determined that the PCR assays for BSI identification must have a very high sensitivity in the test matrix (blood). The *nuc* assay had a LOD of at least 50 CFU per ml blood. This LOD would normally indicate a highly-sensitive assay. However, we determined that a LOD of 50 CFU per ml was insufficient to reliably detect *S. aureus* in the blood of BSI patients. Instead, a LOD<10 CFU per ml was required. We achieved the ultra-high sensitivity of the *sodA* assay by reducing the assay amplicon to the smallest size possible and by using a fully nested-PCR protocol. Even the moderately larger *nuc* amplicon had decreased sensitivity compared to the *sodA* assay. It should be noted that both *nuc* and *sodA* are single copy genes [27] and assay parameters were similar. Thus, the increase in the sensitivity of the *sodA* assay can likely be attributed to the decreased amplicon size. PCR assays designed with relatively small amplicon sizes are known to perform better than larger amplicons, because the PCR kinetics favors smaller amplicons [28], [29], [30], [31].By shortening an amplicon size from 84 bp to 50 bp Sikora et al., [32] improved detection rate of cell-free fetal DNA even in low template samples (early pregnancy). Our study further supports this conclusion and may serve as a guide to designers of BSI detection assays.

The *sodA* assay detected *S. aureus* in BSI patients directly from 1 ml of patient blood with a diagnostic sensitivity of nearly 90%. It is likely that the sensitivity of our assay could be further improved by increasing the sample volume. Our results are particularly remarkable given that our study was not limited to patients with overt sepsis. Septic patients might be expected to have relatively higher concentrations of bacteria in their blood. Rather, we tested all patients who had positive blood cultures for *S. aureus* for any reason. It would be interesting to analyze diagnostic sensitivity according to the disease state of the patients studied. Unfortunately, the nature of our study design (which used de-identified samples) precluded this possibility. A prospective trial to study this question is needed; however, it would likely be very expensive, since most enrolled subjects would be culture negative.

*S. aureus* is one of the most common causes of bloodstream infections. We selected this target organism because of its medical importance and because a relatively large number of patients with *S. aureus* BSI were available at our institution. The SeptiFast assay (Roche, Indianapolis, IN) is the only commercially available method designed to directly detect bacteria from blood. Our methodology performed equally well or better than published reports using SeptiFast assay. Detection concordance for SeptiFast assay compared to blood culture has varied highly for individual studies, ranging from 42% [33] to 80% [14], [34] for the detection of any bacteria. Genomic DNA extraction for SeptiFast assay is reported to be laborious and recent studies have used alternative methods of sample processing to reduce the assay time from 6.54 h to 3.56 h (Regueiro et al., 2011). Our assay demonstrated 89% sensitivity with *S. aureus* and took about 2 h to complete with hands-on time of less than 2 min. This assay format can be easily adapted to detect other bacterial causes of BSI in a uniplex or a multiplex PCR platform.

In conclusion, we have identified the assay parameters required for highly sensitive BSI detection that can detect bacteria causing BSI directly from 1 ml of whole blood within 2 h. Although our study focused on *S. aureus* detection, it is likely to be more widely applicable to detection of other causes of bacterial sepsis. The automatic system which we used can detect six different colors, thus it is currently limited to detecting six different probes and targets in a single assay. However, more highly multiplexed approaches are being developed which can potentially expand the ability of a single PCR reaction to detect and distinguish among hundreds of different targets [35]. If applicable to an automated system, it is possible that our approach could detect all medically relevant causes of BSI in a single test. This approach uses an automated hands-free system that minimizes manual technical errors and potentially reduces cost. These results could also be directly employed for developing a point of care diagnostic system. Our approach may lead to life saving diagnostic assays that rapidly identify the infecting pathogen in patients with BSI and sepsis, enabling more rapid diagnosis and treatment of these life-threatening conditions.

## Supporting Information

Data S1 **PCR parameters.**
(DOCX)

Data S2 **Effect of small and large amplicon assays on LOD using** ***sodA*** **assay as a model.**
(TIF)
