# Supplementary material for: Highly Sensitive Detection of Staphylococcus aureus Directly from Patient Blood
Source: PLoS One. 2012 Feb 17;7(2):e31126. doi: 10.1371/journal.pone.0031126 (PMC3281916; doi:10.1371/journal.pone.0031126)
Supplement: Data S1 — PCR parameters. (DOCX) [file pone.0031126.s001.docx]

| **Assay/ Amplicon size** (Outer/ Inner PCR) | **Primer/Beacon** | **Sequence** |  | **Thermal cycling conditions** | | | |
| --- | --- | --- | --- | --- | --- | --- | --- |
|  |  |  |  | Denaturation | Annealing | Elongation | Number of cycles |
| Nuc-heminested  182bp/128bp | nuc F-outer182 | GCGATTGATGGTGATACGGTT |  |  |  |  |  |
|  | nuc F-Inner128 | AAAATGCAAAGAAAATTGAAGTC | Outer PCR | 95°C 15s | 65°C; 30s | 72°C 15s | 20 |
|  | nuc R-Out/Inn | GCCAAGCCTTGACGAACTAAAGC | Inner PCR | 95°C 15s | 54°C; 30s | 72°C 15s | 45 |
|  | Nuc-molecular beacon | ccgcctACTGATAAATATGGACGTGGaggcgg | |  |  |  |  |
|  |  |  |  |  |  |  |  |
| SodA-nested  161bp/79bp | sodA F-outer161 | CCAATGTAGTCAGGGCGTTT |  |  |  |  |  |
|  | sodA R-outer161 | GGTTGGGCTTGGTTAGTCGT | Outer PCR | 95°C 15s | 55°C; 30s | 72°C 15s | 20 |
|  | sodA F-inner79 | GCGTGTTCCCATACGTCTAAA | Inner PCR | 95°C 15s | 58°C; 30s | 72°C 15s | 45 |
|  | sodA R-inner79 | TTGTGACTACACCAAACCAAGA |  |  |  |  |  |
|  | sodA-molecular beacon | agcgcGGTGTTTTACCCTCAGTTAATGGgcgct | |  |  |  |  |
| 79 bp SodA-non nested | sodAF-79nn | GCGTGTTCCCATACGTCTAAACC | | 95°C 15s | 58°C; 30s | 72°C 15s | 45 |
|  | sodAR-79nn | TTGTGACTACACCAAACCAAGATAAT | |  |  |  |  |

**Data S1. PCR parameters**

Note: For all PCRs initial denaturation was performed at 95°C for 1 min.
