# Supplementary figures and images for: Highly Sensitive Detection of Staphylococcus aureus Directly from Patient Blood
Source: PLoS One. 2012 Feb 17;7(2):e31126. doi: 10.1371/journal.pone.0031126 (PMC3281916; doi:10.1371/journal.pone.0031126)

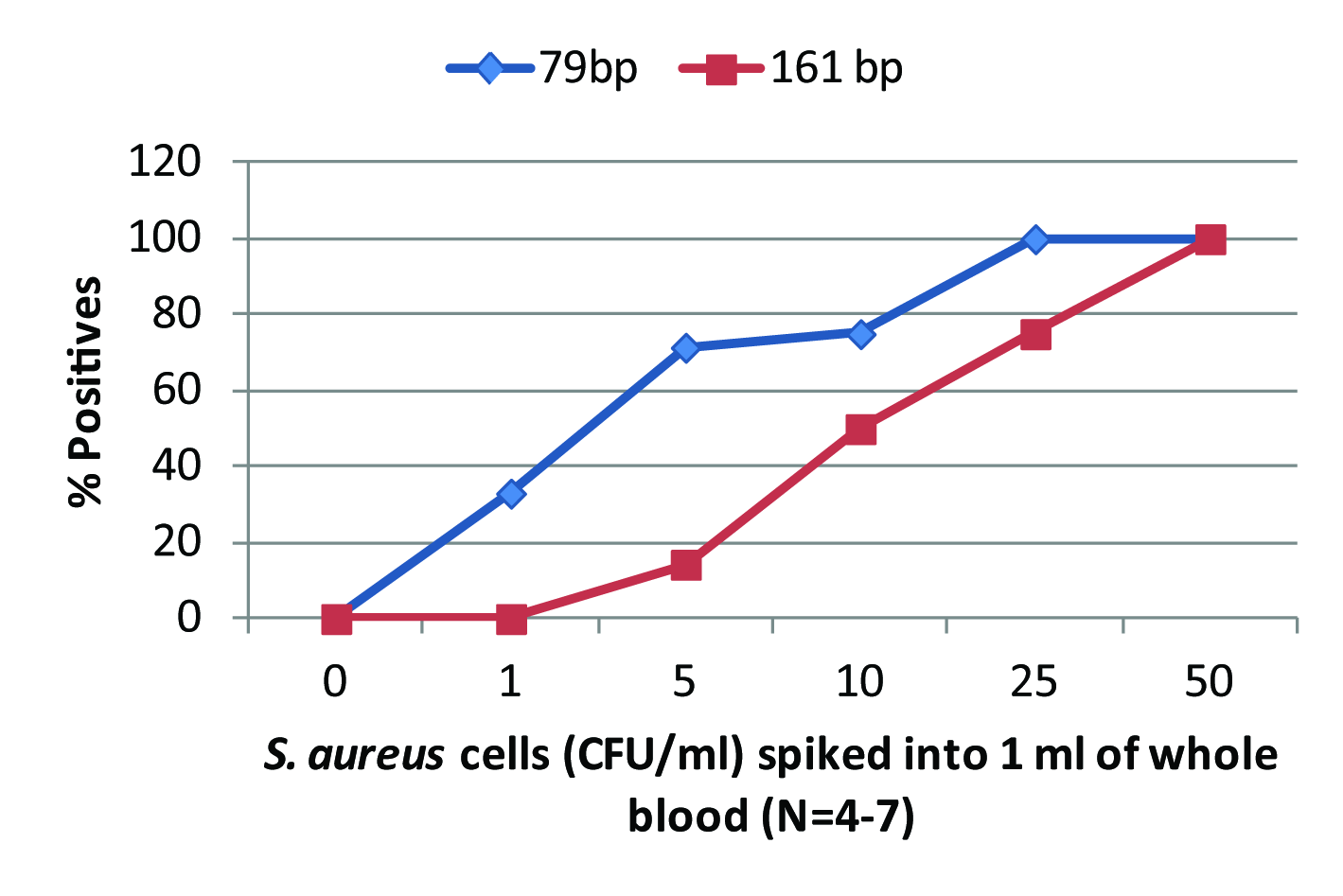

Supplement: Data S2 — Effect of small and large amplicon assays on LOD using sodA assay as a model. (TIF) [file pone.0031126.s002.tif]
